# GRSF1 deficiency in skeletal muscle reduces endurance in aged mice

**DOI:** 10.18632/aging.203151

**Published:** 2021-06-02

**Authors:** Riley K. Driscoll, Linda K. Krasniewski, Samuel G. Cockey, Jen-Hao Yang, Yulan Piao, Elin Lehrmann, Yongqing Zhang, Marc Michel, Ji Heon Noh, Chang-Yi Cui, Myriam Gorospe

**Affiliations:** 1 Laboratory of Genetics and Genomics, National Institute on Aging, National Institutes of Health, Baltimore, MD 21224, USA; 2 Department of Biochemistry, Chungnam National University, Daejeon, Korea

**Keywords:** skeletal muscle aging, GRSF1, RNA-binding protein, mouse aging

## Abstract

GRSF1 is a mitochondrial RNA-binding protein important for maintaining mitochondrial function. We found that GRSF1 is highly expressed in cultured skeletal myoblasts differentiating into myotubes. To understand the physiological function of GRSF1 *in vivo*, we generated mice in which GRSF1 was specifically ablated in skeletal muscle. The conditional knockout mice (Grsf1cKO) appeared normal until 7-9 months of age. Importantly, however, a reduction of muscle endurance compared to wild-type controls was observed in 16- to 18-month old Grsf1cKO mice. Transcriptomic analysis revealed more than 200 mRNAs differentially expressed in Grsf1cKO muscle at this age. Notably, mRNAs encoding proteins involved in mitochondrial function, inflammation, and ion transport, including *Mgarp, Cxcl10, Nfkb2,* and *Sln* mRNAs, were significantly elevated in aged Grsf1cKO muscle. Our findings suggest that GRSF1 deficiency exacerbates the functional decline of aged skeletal muscle, likely through multiple downstream effector proteins.

## INTRODUCTION

The protein GRSF1 (Guanine-rich RNA sequence binding factor 1) is widely distributed in mammalian organs and is predominantly enriched in mitochondria [1]. *GRSF1* mRNA is transcribed from nuclear DNA, but after translation, it is rapidly mobilized to mitochondria [2]. GRSF1 is a critical component of mitochondrial RNA granules, promotes mRNA loading onto mitochondrial ribosomes [2–4], and regulates the mitochondrial localization and function of the long noncoding RNA *RMRP* [5]. GRSF1 is a downstream component of the Wnt signaling pathway [6, 7], and *ex vivo* analysis suggests that GRSF1 is involved in brain development [8]. Notably, GRSF1 expression levels decline in senescent cells, and GRSF1 deficiency results in increased oxidative stress and senescence in cultured cells [1, 9, 10]. These findings suggest that GRSF1 may play an important role in organ aging. However, the *in vivo* function of GRSF1 remains poorly understood.

As one of the largest organs in mammals, skeletal muscle regulates body movement, which requires the production of large amounts of ATP by mitochondria [11]. Hence, skeletal muscle is extremely rich in mitochondria and its function depends on robust mitochondrial function. With advancing age, the progressive loss of skeletal muscle mass and function, known as sarcopenia, leads to reduced muscle strength and diminishes individual mobility, quality of life, and lifespan [12]. In aging skeletal muscle, mitochondria display reduced function, altered morphology, and increased production of reactive oxygen species (ROS), which contribute to a progressive loss of muscle mass and strength [13, 14]. Therefore, skeletal muscle is an ideal organ to study the function of mitochondrial proteins, especially as they influence aging-related processes. In this regard, the involvement of GRSF1 in skeletal muscle aging has not been investigated.

To analyze GRSF1 function in skeletal muscle *in vivo*, we generated skeletal muscle-specific *Grsf1* knockout (Grsf1cKO) mice. The Grsf1cKO mice appeared normal until they were 7-9 months of age. At more advanced ages, however, Grsf1cKO mice showed significantly weaker muscle endurance compared to wild-type (WT) controls. We further identified important alterations in the transcriptomes of Grsf1cKO muscle relative to WT muscle, including differential abundance of mRNAs encoding mitochondrial proteins, proinflammatory factors, and proteins that regulate ion transport. Our results suggest that GRSF1 helps to maintain skeletal muscle function at advanced ages in part by modulating muscle-specific gene expression programs.

## RESULTS

### GRSF1 is highly expressed in differentiating skeletal muscle cells

To begin investigating a possible role for GRSF1 in muscle physiology, we first analyzed the GRSF1 expression pattern in cultured human skeletal muscle myoblast lines AB678 and AB1167 [15]. Human myoblasts were then entered into a myogenic differentiation program that resembled myogenesis during embryonic development and adult muscle regeneration. Specifically, proliferating myoblasts were cultured to high density and the culture media was replaced by media containing horse serum to trigger myogenic differentiation; by 2 days, myotubes began to appear, and were fully established by 3-5 days, as previously described [15].

**Figure 1:**
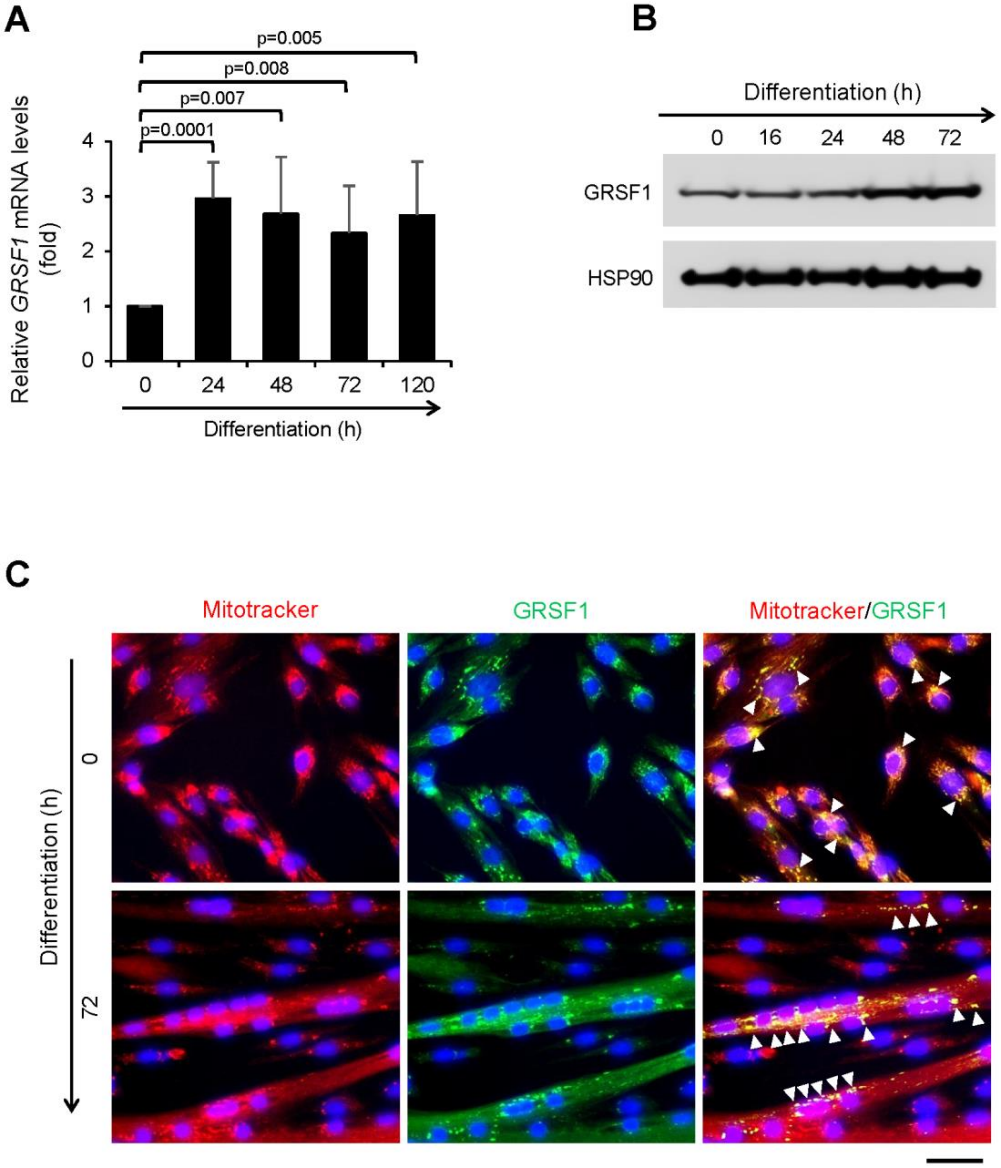
**Expression of GRSF1 across myogenesis.** (**A**) RT-qPCR analysis of *GRSF1* mRNA levels in proliferating (0 h) and differentiating (24-120 h) human myoblasts; n=3. *GRSF1* mRNA levels were normalized to the levels of *GAPDH* mRNA. (**B**) Western blot analysis of the levels of GRSF1 at the indicated times during differentiation; n=2. (**C**) Immunofluorescence detection of GRSF1 (green) and mitochondria (red) in proliferating myoblasts and differentiating myotubes. Arrowheads indicate GRSF1 signals; n=3. Scale bar, 50 μm.

By reverse transcription followed by quantitative polymerase chain reaction (RT-qPCR) analysis, *GRSF1* mRNA was easily detectable in proliferating myoblasts, increasing as myoblasts progressed through myogenesis, and remaining elevated between 24-120 h of myogenic differentiation (Figure 1A). Western blot analysis revealed a rise in GRSF1 protein levels by 48 h of differentiation and sustained high levels of GRSF1 thereafter (Figure 1B). By immunofluorescence microscopy, GRSF1 showed a punctate expression pattern that displayed extensive colocalization with MitoTracker, a mitochondria-specific dye, in both proliferating myoblasts and differentiating myotubes (Figure 1C). These observations in a human cell culture model of myogenesis suggest that GRSF1 may be involved in skeletal muscle development *in vivo*.

### Generation of skeletal muscle specific *Grsf1* knockout mice

**Figure 2:**
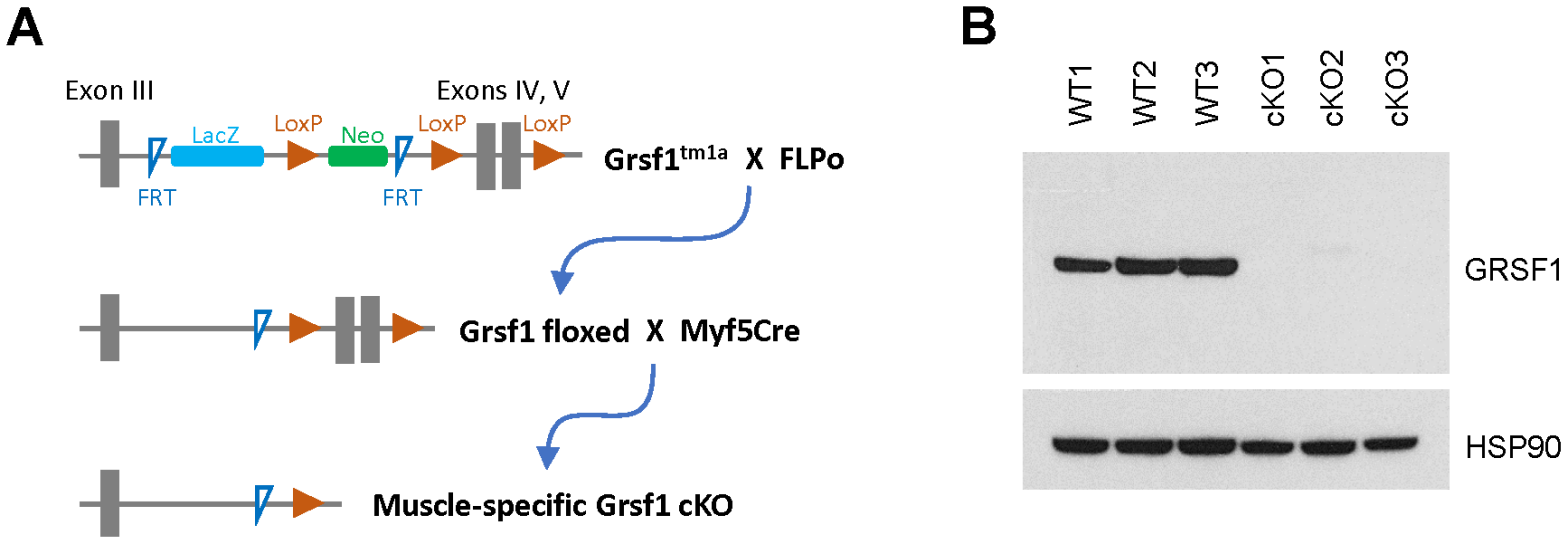
**Generation of skeletal muscle-specific *Grsf1* knockout mice.** (**A**) Schematic of the generation of skeletal muscle specific *Grsf1* knockout mice from the original Grsf1^tm1a^ mice. (**B**) Western blot analysis of the levels of GRSF1 in WT and Grsf1cKO RF muscle.

To analyze GRSF1 function in skeletal muscle *in vivo*, we generated mice in which the *Grsf1* gene was selectively ablated in skeletal muscle. Using heterozygous *Grsf1* mutant mice (Grsf1^tm1a^) from the European Mouse Mutant Archive (EMMA), a LacZ cassette, a Neo cassette, 2 FRT (flippase recognition target) sites, and 3 LoxP (locus of X-over P1) sites were inserted into a *Grsf1* allele (Figure 2A). We then removed the LacZ and Neo cassettes by crossing the Grsf1^tm1a^ mice with FLPo (flippase recombinase transgenic) mice (Figure 2A). In the resulting Grsf1-LoxP mice, exons IV and V of *Grsf1* were floxed. The *Grsf1*-floxed mice were further crossed with Myf5Cre mice to generate skeletal muscle-specific *Grsf1* knockout (Grsf1cKO) mice (Figure 2A). As expected, Western blot analysis confirmed that GRSF1 protein production was almost undetectable in skeletal muscle of the homozygous knockout progeny (Figure 2B). Grsf1cKO mice were viable and fertile, and they appeared healthy (data not shown).

### Grsf1cKO mice showed weak muscle endurance at advanced ages

To understand the effects of *Grsf1* ablation in skeletal muscle, we measured muscle endurance in sex- and age-matched WT control and Grsf1cKO mice at 2-4, 7-9, and 16-18 months of age. The Grsf1cKO mice showed comparable endurance in the treadmill test to that of WT mice until 7-9 months of age (Supplementary Figure 1A). Body weight, lean mass, fat mass and the ratio of lean/fat were similar between WT and Grsf1cKO mice at this age (Supplementary Figure 1B, 1C). These results suggest that Grsf1 is dispensable for skeletal muscle development and maturation through at least 9 months of age.

**Figure 3:**
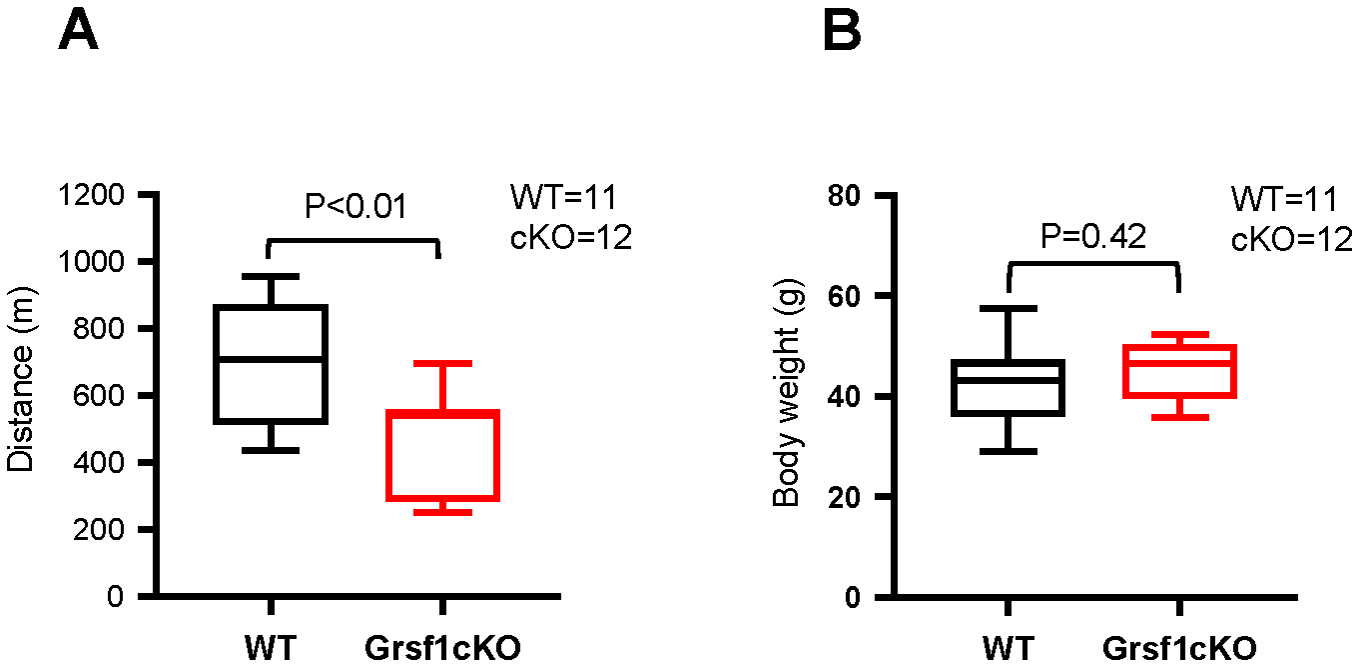
**Grsf1cKO mice show weaker muscle endurance at advanced ages.** (**A**) Treadmill test to assess skeletal muscle endurance in Grsf1cKO and WT mice at 16-18 months of age. (**B**) Body weights of Grsf1cKO and WT mice.

Interestingly, aged (16-18 months old) Grsf1cKO mice showed weaker muscle endurance compared to WT controls (Figure 3A). The Grsf1cKO mice at this age ran about a 30% shorter treadmill distance on average relative to WT controls (Figure 3A). The test was repeated two weeks later with the same mice and the results were comparable. The Grsf1cKO and WT control mice had similar body weights and gross appearances at this age (Figure 3B). Histological analysis showed both normal (Supplementary Figure 2A, upper left panel) and degenerating fibers (arrows in Supplementary Figure 2A, lower left panel) in WT mice at this age, indicating normal progression of natural muscle aging, with similar morphological features in Grsf1cKO skeletal muscle (Supplementary Figure 2A, right panels). These results suggest that the impact of losing GRSF1 on skeletal muscle function in mice may not be apparent until later in life. The ages of the mice tested at the time of analysis are listed in Supplementary Figure 1D.

### Differentially expressed mRNAs in WT vs Grsf1cKO muscle by expression profiling analysis

**Figure 4:**
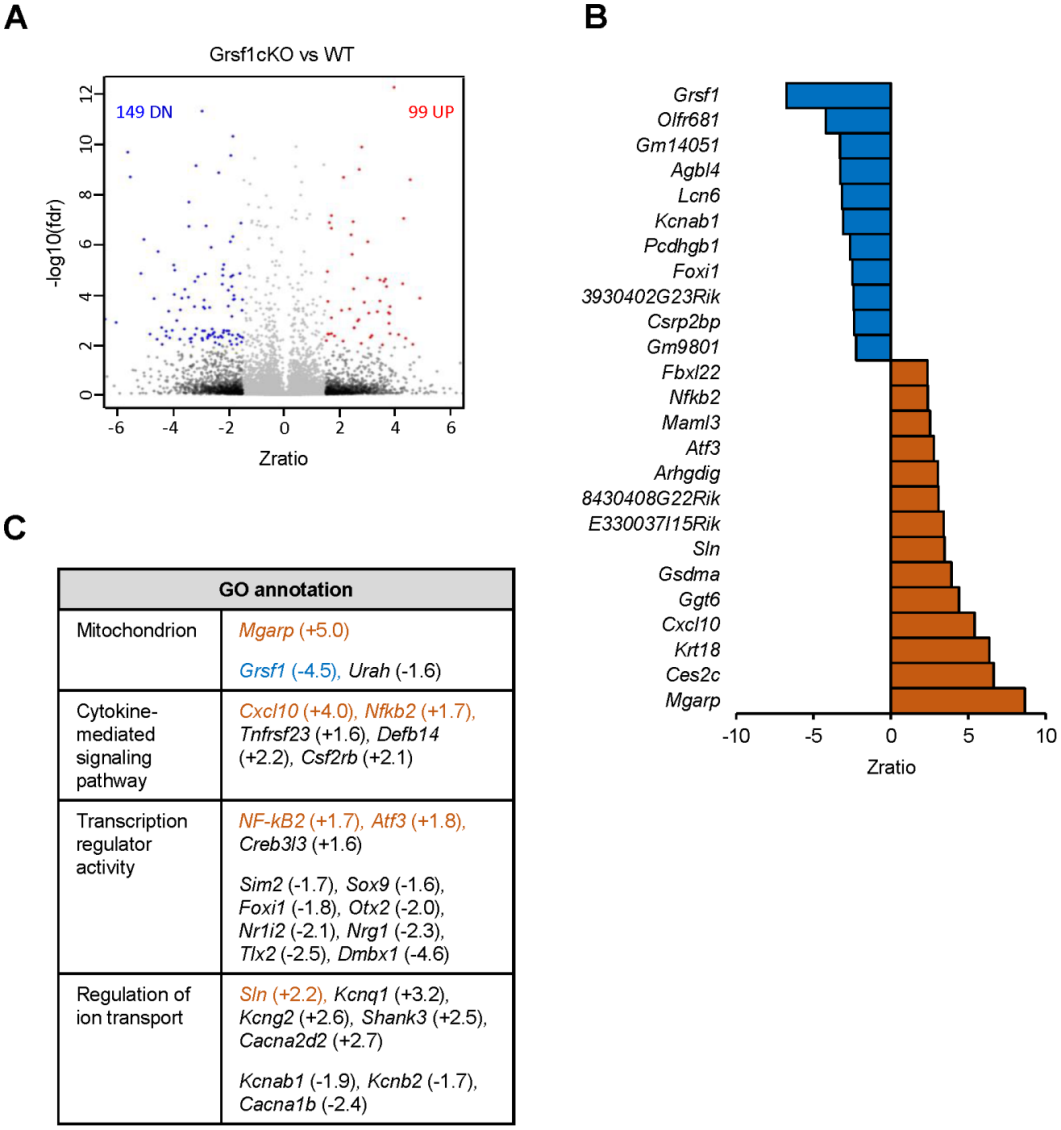
**Microarray analysis of RNAs differentially expressed in skeletal muscle from Grsf1KO vs WT mice.** (**A**) Volcano plot showing differentially expressed RNAs (including both mRNAs and long noncoding RNAs detected by probes in Agillent microarrays) in the Grsf1cKO vs WT RF muscles; n=3 mice for each genotype. (**B**) List of Top 25 RNAs significantly less abundant (top, blue) or significantly more abundant (bottom, brown) in Grsf1KO relative to WT mouse muscle. (**C**) GO annotations of functional groups significantly different in Grsf1cKO muscles. Numbers in parentheses show fold-changes. (+), upregulated in Grsf1cKO vs WT; (-), downregulated in the Grsf1cKO vs WT. Brown: upregulated mRNAs confirmed by RT-qPCR analysis. Blue: downregulated mRNA confirmed by RT-qPCR analysis.

**Figure 5:**
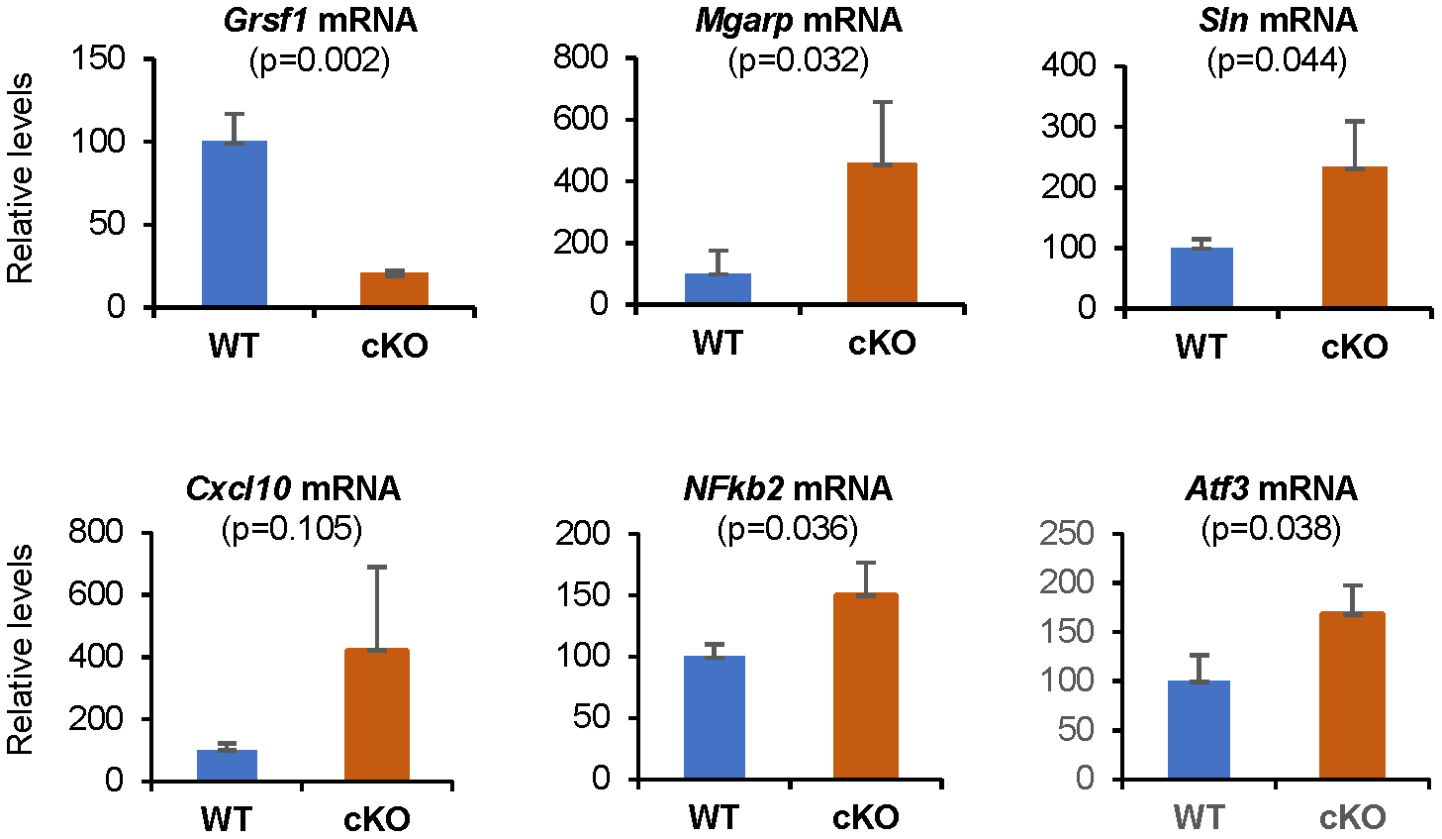
**RT-qPCR validation of microarray results.** Levels of *Grsf1* mRNA as well as *Mgarp*, *Sln*, *Cxcl10*, *Nfkb2*, and *Atf3* mRNAs in muscle from Grsf1cKO and WT mice; n=3 mice for each genotype. The levels of the mRNAs shown were normalized to the levels of *Gapdh* mRNA in each sample.

To understand the molecular changes accompanying the decline of muscle endurance in aged Grsf1cKO mice, we carried out gene expression analysis in skeletal muscle harvested from Grsf1cKO mice and WT mice by using microarrays. We isolated total RNA from a quadricep muscle, the rectus femoris (RF), which contains both slow and fast muscle fibers [16]. Three 17-month-old Grsf1cKO mice and three age- and sex-matched WT control mice were analyzed (GEO accession number GSE172311). We found 99 transcripts elevated and 149 transcripts decreased in abundance in the Grsf1cKO muscle compared to WT controls using these criteria: fold-change > 1.5, FDR < 0.2, and p < 0.01 (Figure 4A, and Supplementary Table 1 for full list). The top 25 RNAs significantly altered in expression levels in the Grsf1cKO muscle are shown in Figure 4B. As expected, *Grsf1* mRNA abundance was dramatically lower in the Grsf1cKO muscle (Figure 4B, Figure 5, and Supplementary Table 1), but many RNAs also showed increased abundance in Grsf1KO muscle samples. GO annotation analysis using g:Profiler further identified several functional clusters from the significantly altered RNAs, including mitochondrial proteins, inflammatory proteins, ion transporters, and transcription factors (Figure 4C). This group included the hypoxia-inducible mitochondrial transcript *Mgarp* mRNA [17] and inflammation-related transcripts *Cxcl10* and *Nfkb2* mRNAs, all elevated in the Grsf1cKO muscle (Figure 4C). Sarcolipin, a key regulator of the sarcoplasmic reticulum calcium pump SERCA, encoded by the *Sln* mRNA [18], was also significantly elevated in the Grsf1cKO muscle (Figure 4B, 4C).

We validated the microarray data by performing RT-qPCR analysis (Figure 5), and confirmed significant changes in the expression levels of several transcripts, including *Grsf1*, *Mgarp*, *Sln*, *Cxcl10*, *Nfkb2*, and *Atf3* mRNAs. *Mgarp* mRNA, almost undetectable in WT controls, was significantly more abundant in Grsf1cKO muscles. *Cxcl10* mRNA levels were elevated in all 3 Grsf1cKO mice analyzed, but to varying degrees, ranging from two to seven-fold, which yielded a *p* value of 0.105 (Figure 5, *Cxcl10* mRNA), despite plausible biological significance.

We further analyzed the expression levels of several markers of tissue aging and cell senescence in both Grsf1cKO and WT control muscles (including *Il6*, *Tnf*, *ND6*, *p15*, *p16*, and *p21* mRNAs) using RT-qPCR analysis and one to three different probe/primer sets for each mRNA (Materials and Methods), with no significant expression changes indicated (data not shown).

### Subtle changes in ROS production in the Grsf1cKO muscle

No obvious inflammatory cell infiltration or regenerating muscle fibers were found in WT or Grsf1cKO mice by histological analysis (Supplementary Figure 2A). To assess ROS production in the Grsf1cKO and WT control mice, we carried out dihydroethidium (DHE) staining. DHE fluorescence in the cell nuclei indicates the presence of ROS [19]. Comparable DHE positive signals were seen in the nuclei of both WT and Grsf1cKO in 17-month-old mice muscle (Supplementary Figure 2B, middle and right panels), suggesting similar ROS production in the Grsf1cKO and WT control mice. As a DHE-positive control, we included a sample from a naturally aged (30-month-old) WT mouse [20, 21] (Supplementary Figure 2B, left panels). These results suggest that, at least in conditions of normal development in adult mice, loss of GRSF1 does not influence oxidative stress levels in skeletal muscle. Taken together, our results indicate that loss of GRSF1 in mouse skeletal muscle did not cause overt changes in morphology or ROS production with advancing age, but it did change gene expression programs and impaired muscle endurance.

## DISCUSSION

The function of GRSF1 has been extensively examined in cultured cells and has shown that GRSF1 plays a critical role in mitochondrial function and cell senescence [1, 8, 9]. To understand the physiological function of GRSF1 *in vivo*, we knocked out the *Grsf1* gene in mice, specifically in the mitochondria-rich skeletal muscle. Although GRSF1 was highly expressed in differentiating myocytes and mature skeletal muscle, Grsf1cKO mice appeared normal, and their muscle endurance was comparable to that of WT control mice until 7-9 months of age, before the appearance of the aging phenotype in normal mice. However, we observed a decline in muscle endurance at 16-18 months of age in the GRSF1cKO mice, where natural muscle degeneration was observed histologically in WT mice (Supplementary Figure 2A). We posit that GRSF1 deficiency may potentiate the aging-associated decline in skeletal muscle function.

The reduction of endurance in Grsf1cKO muscle was accompanied by differential expression of several mRNAs, including some that encoded mitochondrial proteins, inflammatory proteins, ion transporters, and transcription factors (*Mgarp*, *Sln*, *Cxcl10*, *Nfkb2*, and *Atf3* mRNAs). The hypoxia-inducible *Mgarp* mRNA was significantly elevated in the Grsf1cKO muscle. The encoded protein, MGARP, regulates mitochondria distribution in neurons [17], and overexpression of MGARP was detrimental for mitochondrial structure [22]. Loss of GRSF1 might affect mitochondrial structure and/or function by overexpressing MGARP in skeletal muscle, although this remains to be tested. We also found that the mRNA encoding Sarcolipin (SLN), an inhibitor of the sarcoplasmic reticulum Ca^2+^ ATPase SERCA, was significantly elevated in the Grsf1cKO muscle. Sarcolipin is abundantly expressed in Duchenne muscular dystrophy [23], and promotes an aging-associated muscle cell fibrosis [24]. Additionally, excess SLN was found to impair contractile function of slow muscle fibers by inhibiting calcium uptake by SERCA in the sarcoplasmic reticulum [25]. It is possible that increased *Sln* mRNA may similarly contribute to the decline in skeletal muscle endurance in Grsf1cKO mice. In addition, Grsf1cKO led to a rise in mRNAs encoding the pro-inflammatory proteins CXCL10 and NFKB2 (NF-κB2) in aged mice. CXCL10 is involved in inflammatory diseases [26], and in inflammatory myopathies [27], and CXCL10 levels rise in response to muscle damage [28]. The *Atf3* mRNA, encoding the transcription factor ATF3, was increased in Grsf1cKO mice; ATF3 levels rise in response to muscle damage and suppresses the production of inflammatory proteins that accumulates with damage [29, 30]. The validation of the aging-associated microarray changes supports the notion that altered gene expression program, including those of key factors involved in maintaining skeletal muscle energy, proinflammatory factors, and ion transporters might underline the decline of muscle endurance in old Grsf1cKO muscle.

GRSF1 was shown to suppress oxidative stress in cultured cells [1], and a recent report suggested that GRSF1 may suppress myogenesis by reducing mitochondrial ROS production [31]. However, ROS production in aged Grsf1cKO muscle was comparable to that of aged WT mice, even though only Grsf1cKO mice displayed reduced muscle endurance. Although we recognize that subtle changes in ROS levels in Grsf1cKO muscle may contribute to our findings, or that changes in ROS before our analysis may have influenced the results, our observations suggest that differences in ROS levels did not significantly influence the skeletal muscle phenotype observed in aged Grsf1cKO mice.

Overall, the function of GRSF1 in skeletal muscle appeared to be moderate, despite our prediction that loss of GRSF1 might deeply impair muscle development and function, based on our earlier findings in cultured cells [1, 2]. This modest *in vivo* effect suggests that there are redundant or compensatory mechanisms that prevent catastrophic damage from GRSF1 loss in aging muscle, and that identifying such factors might be of therapeutic benefit in diseases caused by impaired function of muscle mitochondria and impaired muscle regeneration.

## MATERIALS AND METHODS

### Differentiation of myoblasts in culture

Immortalized human myoblasts AB678 and AB1167, which were previously described [15], were cultured in growth medium (equal volume mixture of Hamm's F10 media with 20% FBS and Promocell Skeletal Muscle Cell Growth Medium). Myoblasts were induced to differentiate for up to 120 h by growth to confluency and replacement of the growth medium with differentiation medium (DMEM with 2% horse serum). For RT-qPCR, Western blot, and immunofluorescence analyses, cells were harvested at the times indicated after initiating differentiation (Figure 1).

### Reverse transcription (RT) and real-time quantitative (q) PCR analysis

For RT-qPCR analysis, cultured myoblasts were harvested at 0, 24, 48, 72 and 120 hours after the beginning of differentiation. Total RNA from cultured cells was isolated using the Direct-zol™ RNA MiniPrep kit (ZymoResearch), which includes a digestion step using DNase I. After reverse transcription (RT) using Maxima reverse transcriptase following the manufacturer’s protocol (Thermo Fisher Scientific), real-time quantitative (q)PCR amplification was carried out using SYBR green and the following specific primers (forward and reverse in each case): GRSF1-F, CAGGGAGCTGATTGCTGAATAand GRSF1-R, ACGCATAAAGGGACACATACTC to amplify *GRSF1* mRNA, and GAPDH-F, TCTGCTCCTCCTGTTCGAC and GAPDH-R, ACGACCAAATCCGTTGACTC to amplify *GAPDH* mRNA. Relative RNA levels were calculated after normalizing to GAPDH mRNA using the 2^-ΔΔCt^ method.

For RT-qPCR assays of mouse tissue, total RNA was isolated from frozen RF muscles with Trizol (Ambion, 15596-026) and the Purelink RNA mini kit (Ambion, 12183018A) followed by on-column Purelink DNase (Ambion, 12185-010) treatment. RT was performed by synthesizing cDNAs from the Grsf1cKO and WT control RNAs with the Superscript IV VILO Master Mix (Invitrogen, 11756050) and qPCR amplification was carried out using ready-to-use Taqman probe/primer sets (Applied Biosystems) to detect *Grsf1* mRNA (Mm00618578_g1), *Mgarp* mRNA (Mm00471236_m1), *Cxcl10* mRNA (Mm00445235_m1), *Nfkb2* mRNA (Mm00479807_m1), *Sln* mRNA (Mm00481536_m1), *Atf3* mRNA (Mm00476033_m1), *Il6* mRNA (Mm00446190_m1; Mm00446191_m1), *ND6* mRNA [Mm04225325_g1; qMmuCED0041184 (SybrGreen); qMmuCED0061740 (SybrGreen)], *Tnf* mRNA (Mm00443258_m1; Mm99999068_m1; Mm00443260_g1), *p15* mRNA (Mm00483241_m1), *p16* mRNA (Mm00494449_m1), and *p21* mRNA (Mm04205640_g1). Three biological replicates were used for the WT and Grsf1cKO genotypes and assayed in triplicate. Quantification of PCR products was performed using standard curves for each probe/primer set and RNA from WT muscles, except in the case of *Mgarp* mRNA, which was very low in WT muscle, so the Grsf1cKO RNA was used here. Reactions were normalized to *Gapdh* mRNA levels, and the data were analyzed for significance using Student's *t*-test.

### Western blot analysis

For Western blot analysis, protein lysates were prepared from cultured myoblasts using RIPA buffer (Thermo Fisher Scientific, 25 mM Tris-HCl pH 7.6, 150 mM NaCl, 1% NP-40, 1% sodium deoxycholate, 0.1% SDS). Protein lysates were also prepared from rectus femoris (RF) muscles that were harvested under a dissection microscope from euthanized 4-month-old Grsf1cKO and WT control mice. Muscles were then homogenized in RIPA buffer containing a protease inhibitor cocktail, PMSF, and sodium orthovanadate (Santa Cruz, SC-24948). Homogenates were centrifuged at 10,000 × *g* for 10 min, and the supernatants were collected. The protein concentration of the supernatants was quantified with the BioRad Protein Assay Dye Reagent (Bradford reagent, 5000006). Protein aliquots (30 μg from each sample) were resolved on 1.5-mm thick, 4-12% gradient bis-tris polyacrylamide gels (Invitrogen, NPO322PK2) and blotted onto nitrocellulose membranes (Bio-Rad, 1704270). Membranes were blocked in 5% milk (Bio-Rad, 1706404xtu) for 1 h at 25° C, and the primary and secondary antibodies were diluted in this same solution. Membranes were incubated fo 16 h with anti-GRSF1 antibody (Sigma Aldrich, #HPA036985, 1:1000; Abcam, ab205531, 1:3000) or anti-HSP90 antibody (Santa Cruz, SC-13119, 1:10000 or SC-101494, 1:2000) at 4° C for 16 h. An HRP-conjugated anti-rabbit secondary antibody (Kindle Biosciences, R1005, 1:10,000) was incubated with the membrane for 1 h at 25° C. Positive bands were visualized using the ECL (enhanced chemiluminescence) reagent (Millipore, WBKLS0500).

### Generation of skeletal muscle-specific Grsf1 knockout mice

The generation and analysis of the mutant mice were approved by the Animal Care and Use Committee of the National Institute on Aging. C57BL/6N-A^tm1Brd^ Grsf1^tm1a(EUCOMM)Wtsi^/WtsiIeg (Grsf1^tm1a^) mice harboring a mutant allele in the *Grsf1* locus were purchased from the European Mouse Mutant Archive (EMMA, EM:10950). In the mutant allele, exons 4 and 5 of *Grsf1* were floxed by two LoxP sites (Figure 1A). Additionally, a LacZ and a Neo cassette, sandwiched by two FRT (Flippase recognition target) sites were inserted into intron 3. To generate conditional Grsf1-LoxP mice, we deleted the LacZ and Neo cassettes by crossing Grsf1^tm1a^ mice with FLPo mice (Stock No: 007844, Jackson Laboratory) expressing the Flippase widely under the Gt(ROSA)26Sor promoter. The resultant Grsf1-LoxP (Grsf1 floxed) mice were crossed further with the Myf5Cre mice (Stock No: 007893, the Jackson Laboratory) to generate skeletal muscle-specific *Grsf1* knockout (Grsf1cKO) mice (Figure 1A). Genotyping was done by Transnetyx (Cordova, TN) following protocols from EMMA and Jackson Laboratories.

### Muscle endurance test and whole-body composition analysis

Treadmill tests were carried out using the Exer 3/6 Animal Treadmill (Columbus Instruments), which was maintained with an 8.75% (5-degree) incline and 3 Hz, 0.15 mA, 163 V electric stimulus. For training, mice were subjected to the following scheme prior to the test: run 5 min at 5 m/min speed, followed by 5 min at 10 m/min, and 5 min at 5 m/min (first day); run 5 min at 5 m/min, followed by 5 min at 10 m/min, 5 min at 15 m/min, and 5 min at 5 m/min (second day); repeat the second day protocol (third day). For the test, mice were allowed to run for 5 min at 5 m/min, followed by 20 min at 10 m/min, 20 min at 15 m/min, 20 min at 20 m/min, 20 min at 25 m/min, and to exhaustion at 30 m/min. The test was ended and mice considered exhausted after they made contact with the electric stimulus 3 times within a period of 5 seconds. Whole body composition analysis was done in live animals using a Bruker Minispec Lf50 Body Composition Analyzer according to the manufacturer’s protocol.

### Gene expression profiling by microarrays

Total RNA was isolated from frozen RF muscles from three 17-month-old Grsf1cKO and three WT control mice using Trizol (Ambion, 15596-026) and the Purelink RNA mini kit (Ambion, 12183018A) followed by on-column Purelink DNase treatment (Ambion, 12185-010). RNA concentration and quality were assessed by using a NanoDrop (ThermoFisher, Waltham, MA, USA) and on the Agilent Bioanalyzer RNA 6000 Chip (Agilent, Santa Clara, CA). Two-hundred ng total RNA was labeled using the Agilent Low-Input QuickAmp Labeling Kit, and purified and quantified following the manufacturer’s recommendations. A total of 600 ng Cy3-labeled cRNA was hybridized for 17 h to Agilent SurePrint G3 Mouse GE v2 8x60K microarrays (G4852B, Design ID 074809), containing 34,134 mRNA and 4,578 lncRNA probes. Following post-hybridization rinses, arrays were scanned using an Agilent SureScan microarray scanner at 3 micron resolution, and hybridization intensity data extracted from the scanned images using Agilent’s Feature Extraction Software. Individual sample signal intensities were sample-wise quantile normalized to obtain consistent distributions prior to obtaining log2-transformed *z*-scores and performing z-test statistics. Sample clustering, correlation, and principal component analysis (PCA) were used to assess data quality and identify possible outlier sample(s). Filtering at the sample level probe *p*-value enabled calculation of a Present-Absent Index (PA call) to identify globally detected probe sets, which were used for analysis of statistically differentially expressed genes. A global, one-way ANOVA test grouped on the sample group was used to filter out the most diverse probes in the sample set, and to ensure that the *inter*-sample group variance was smaller than the *intra*-sample group variance. Pairwise z-test comparisons for each comparison were performed to select probes with (1) an absolute *z*-ratio ≥ 1.5, (2) z-test *p*-value ≤ 0.01, (3) fdr ≤ 0.20, and (4) an average z-normalized gene expression in the comparison set > 0. Significant gene sets for each comparison were further studied using Ingenuity Pathway Analysis (IPA) suite and the gene expression deferential values are used to do gene set enrichment analysis against Gene Ontology and Pathway gene sets for functional analysis results by the PAGE algorithm. Raw and z-score normalized hybridization intensity data have been deposited with GEO under accession number GSE172311.

### Histology and ROS detection

For histological analysis, RF muscles collected from Grsf1cKO and WT mice were immediately frozen in liquid nitrogen-chilled isopentane (Sigma-Aldrich, M32631) for 45 sec and stored at -80° C until use. Tissues were sectioned at -20° C at 5-8 μm onto silane-pre-treated slides (Sigma-Aldrich, S4651), dried at 25° C for >30 min and kept at -20° C until use. For general histology, frozen sections were stained with Mayer’s Hematoxylin solution (Sigma-Aldrich, MHS128) and Eosin Y solution (Sigma-Aldrich, HT1102128) according to the manufacturer’s recommendations.

ROS levels were assessed by using a dihydroethidium (DHE) fluorescent staining protocol adapted from a previous publication [32]. The frozen RF sections from three 17-month-old Grsf1cKO mice and three 17-months-old WT control mice were fixed in cold acetone (-20° C) for 3 min and then rinsed in 1X phosphate buffered saline (PBS, pH 7.4) three times for 5 min each time. A DHE stock solution (5 mM, Invitrogen, D23107) was diluted to 50 μM in 1X PBS, and this working solution was applied to the sections for 30 min at 37° C in a dark, humidified container. Slides were rinsed in 1X PBS three times and covered with a coverslip with SlowFade™ Diamond Antifade Mountant (Invitrogen, S36963). Images were acquired using a DeltaVision Microscope System (Applied Precision, 20x magnification) equipped with a 542-nm (TRITC) excitation laser. Heart sections from a 30 month old WT mouse were used as a positive control.

### Immunofluorescence

For immunostaining of differentiated muscle cells, cells were incubated with MitoTracker (Invitrogen, #M7512) for 30 min at 50 nM, followed by fixation in 4% paraformaldehyde in PBS for 10 min and permeabilization in 0.5% TritonX-100 in 1X PBS for 5 min. Slides were then incubated with a GRSF1 antibody (Sigma Aldrich, HPA036985, 1:200) overnight at 4° C, followed by incubation in an Alexa Fluor 594 donkey anti-rabbit secondary antibody (Invitrogen, A21207, 1:1000) for 45 min at 25° C. Signals were visualized with the same DeltaVision microscope system described above but at 10x magnification and using 390-nm (DAPI) and 475-nm (FITC) excitation lasers in addition to the TRITC laser.

## Supplementary Material

Supplementary Figures

Supplementary Table 1
