# Supplementary material for: GRSF1 deficiency in skeletal muscle reduces endurance in aged mice
Source: Aging (Albany NY). 2021 Jun 2;13(11):14557–70. doi: 10.18632/aging.203151 (PMC8221292; doi:10.18632/aging.203151)
Supplement: Supplementary Figures [file aging-13-203151-s001.pdf]

SUPPLEMENTARY FIGURES

A

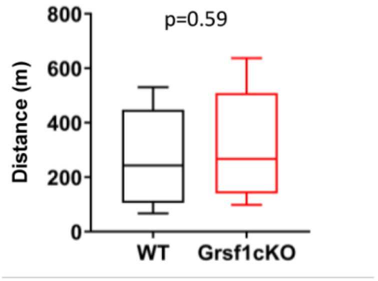

B

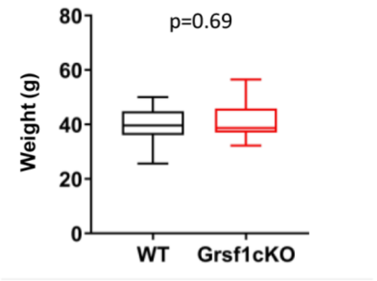

C

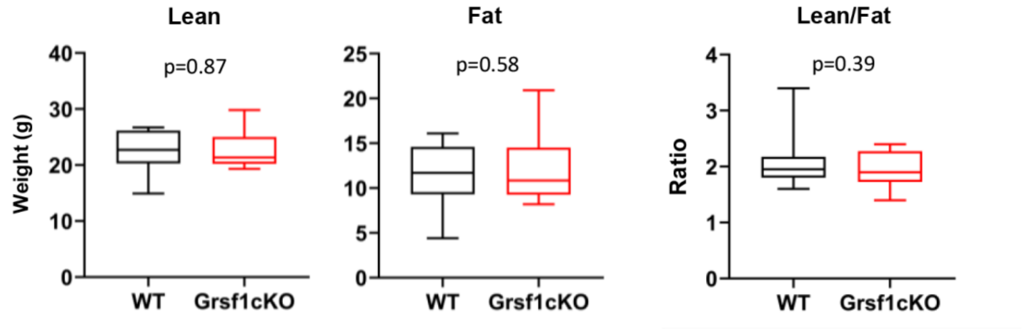

D

WT ID	Age (mo)	cKO ID	Age (mo)
CYC16318	16.5	CYC16291	16.6
CYC17031	16.8	CYC16775	16.7
CYC15415	17.2	CYC16185	17.3
CYC16190	17.3	CYC16131	17.3
CYC16142	17.3	CYC16130	17.4
CYC16145	17.3	CYC16117	17.5
CYC16128	17.4	CYC16116	17.5
CYC16118	17.5	CYC16098	17.6
CYC16097	17.6	CYC16090	17.6
CYC16584	17.9	CYC16588	17.9
CYC15308	18	CYC16582	17.9
		CYC16019	17.9
**Average age**	**17.3**	**Average age**	**17.4**

Supplementary Figure 1. Endurance test and whole-body composition analysis at 7-9 months of age. (**A**) Treadmill tests to assess muscle endurance in Grsf1cKO and WT mice at 7-9 months of age. (**B**) Body weights at 7-9 months of age. (**C**) Lean mass, fat mass, and lean/fat ratios in Grsf1cKO and WT mice. (**D**) Table listing ID numbers and ages of each mouse analyzed.

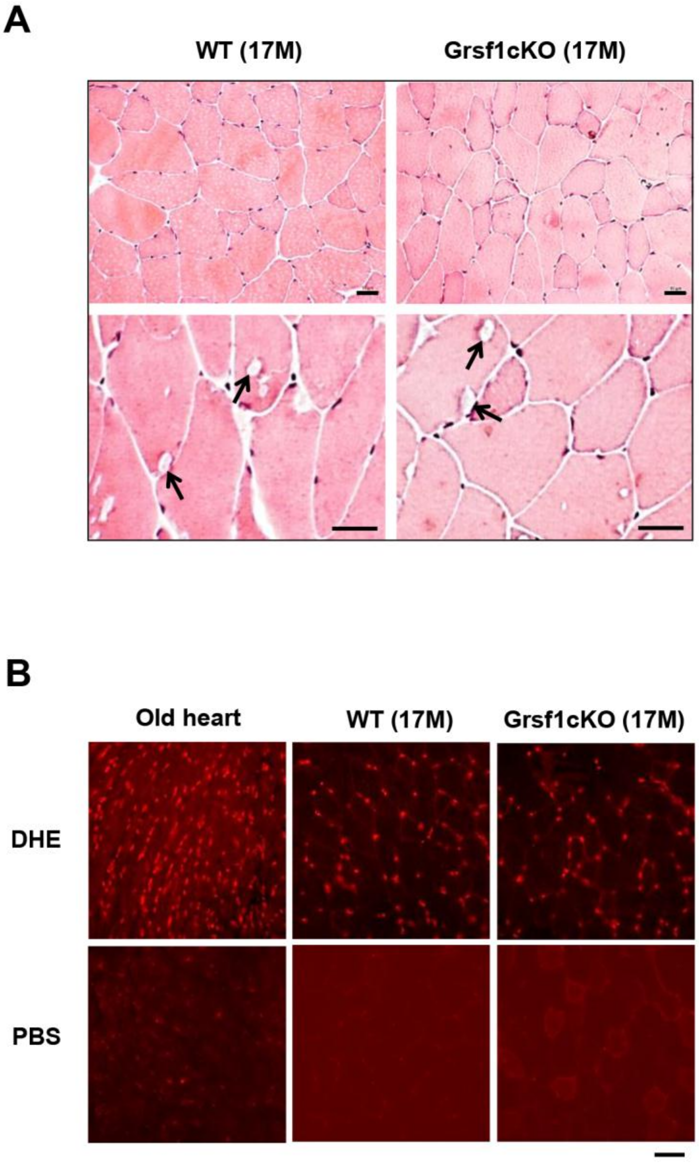

Supplementary Figure 2. Histological analysis and ROS detection. (**A**) Grsf1cKO and WT muscle histology showing normal morphology (upper panels) and the presence of marks of degeneration (arrows in lower panels) at 17 months of age. (**B**) DHE staining showed comparable ROS accumulation in Grsf1cKO and WT muscles (middle and right panels) in 17-month-old (17M) mice. 'Old Heart', DHE staining of 30-month-old heart muscle from normally aged mouse, as a positive control. Scale bars, 50 µm.
